# Phages Bind to Vegetative and Spore Forms of *Paenibacillus larvae* and to Vegetative *Brevibacillus laterosporus*

**DOI:** 10.3389/fmicb.2021.588035

**Published:** 2021-01-26

**Authors:** T. Scott Brady, Charles R. Roll, Jamison K. Walker, Christopher P. Fajardo, Donald P. Breakwell, Dennis L. Eggett, Sandra Hope

**Affiliations:** ^1^Department of Microbiology and Molecular Biology, Brigham Young University, Provo, UT, United States; ^2^Department of Statistics, Brigham Young University, Provo, UT, United States

**Keywords:** American Foulbrood, bacteriophage, phage therapy, *Paenibacillus larvae*, bacterial spores, phage binding, spore binding, Brady Binding Assay

## Abstract

*Paenibacillus larvae* is the causative agent of American Foulbrood (AFB), the most destructive bacterial infection in honeybees. Even antibiotic-sensitive strains of *P. larvae* can produce recurrent AFB months to weeks post-antibiotic treatment due to the survival of bacterial spores. Recently, phages that infect *P. larvae* have been shown to effectively combat AFB in the field. Here, we present evidence that phages not only bind to vegetative *P. larvae* but also bind to *P. larvae* spores. Spore binding was observed in the results of three specific experiments: (1) bacteria counted by flow cytometry generated quantitative data of FITC-labeled phages that were bound to vegetative bacteria as well as those bound to spores, (2) electron microscopy captured images of phages bound to the surface of spores in both horizontal and vertical positions, and (3) phages incubated with *P. larvae* spores bound to the spores and created plaques in vegetative bacteria under conditions not conducive to spore activation, indicating that binding to spores is reversible and that the phages are still active. Identification of phages with reversible spore-binding capability for use in phage therapy may improve treatment of sporulating bacterial infections.

## Introduction

The spore forming bacterium *Paenibacillus larvae* causes American Foulbrood (AFB) in honeybees. An AFB outbreak begins when there are too many *P. larvae* spores in the honey crop of a nurse bee to be cleared naturally and spores get passed to honeybee larvae (Lindstrom et al., 2008). In the larval intestinal tract, *P. larvae* spores germinate to become vegetative bacteria capable of producing toxins that liquefy the honeybee larvae (Djukic et al., 2014). The resulting degraded larvae become laden with *P. larvae* spores which are then tracked to other larvae in the hive by nurse honeybees (Ratnieks, 1992). The disease spreads quickly within a hive, taking just several days from initial infection to decimation of a colony (Chantawannakul and Dancer, 2001; Genersch, 2010). After the colony collapses, other colonies may rob the contaminated honey and further spread AFB via spores with an 80% transmission rate during an outbreak in an apiary (Brady et al., 2017).

Due to their host specificity, phages can target and destroy pathogenic bacteria while leaving commensal bacteria alone because, most typically, a phage is limited to a narrow range of strains of the same species (Merrill et al., 2014; Cresawn et al., 2015; Brady et al., 2017; Hatfull, 2018). The narrow host specificity often requires the use of a phage cocktail (more than one phage) in order to target different strains of the same species (Brady et al., 2017; Dedrick et al., 2019; Corbellino et al., 2020). Phage therapy with an appropriate cocktail is an effective treatment option for active AFB infections, demonstrating a 100% recovery and prevention rate in treated hives (Brady et al., 2017). Furthermore, hives treated with *P. larvae* phages experienced no reinfection of AFB, which may indicate that the phage treatment also neutralized *P. larvae* spores (Brady et al., 2017).

We hypothesize that the ability of *P. larvae* phages to prevent reinfection of AFB as observed by Brady et al. (2017), is due to the *P. larvae* phages’ ability to bind to bacterial spores. To obtain supportive data for this hypothesis, we determined to identify whether *P. larvae* phages could, in fact, bind to spores and whether or not such phages would still be capable of infection. We report that flow cytometry data indicated statistical significance in support of spore-binding, that electron micrographs captured phages attached to the side of spores, and that the phages that bind to *P. larvae* spores were still active and could subsequently infect vegetative bacteria. Another finding of our phage-binding studies was that *P. larvae* phages also bound to vegetative *Brevibacillus laterosporus* at a statistically higher amount than to another unrelated bacterium. *B. laterosporus* is a Firmicutes bacterium also found in honeybees. The *P. larvae* phage used in our studies could not productively infect *B. laterosporus*, so binding of the phages to *B. laterosporus* may indicate a survival mechanism for phages between active AFB infections.

## Materials and Methods

### Spore Generation and Extraction

Overnight cultures of *P. larvae* ATCC 9545 grown in 1/2 × liquid porcine brain heart infusion (PBHI) (Acumedia, Lansing, MI, United States) media were grown in a shaking incubator at 37°C. The optical density of the culture at 600 nm was used to estimate the number of cells per milliliter as we have reported previously (Brady et al., 2017). A total of approximately 10^4^ bacterial cells were spread onto tryptic soy agar plates with glass beads and allowed to incubate at 37°C and 5% CO_2_ for 8 days. Incubated plates were doused with five ml of cold sterile ddH_2_O and allowed to sit for 15 min. Colonies on the plates were gently scraped off the plate and into suspension with sterile loops. The suspensions from eight plates were combined into a 50 ml tube and centrifuged at 12,000 × *g* for 20 min. Supernatant was poured off and the pellet was resuspended in 40 ml of sterile ddH_2_O and centrifuged again as a wash step. The pellet was washed two more times. After the last wash step, the pellet was resuspended in 80% EtOH.

Spores were removed from the ethanol immediately prior to any experiment. Spores suspended in ethanol were centrifuged at 12,000 × *g* for 5 min to pellet the spores and the supernatant containing alcohol and the lysed debris from dead vegetative cells were discarded with the supernatant. The pellet was washed three more times using sterile 1/2 × PBHI broth and then suspended to a concentration of 10^4^ colony forming units (CFU) per ml. Spore purity was also confirmed using the Schaeffer-Fulton staining method: briefly, samples were heat fixed, stained with 5% malachite green for 5 min over heat, and counterstained with 0.2% safranin (Schaeffer and Fulton, 1933). Spore viability and purity tests were done with samples in triplicate. The malachite green and safranin staining was done to verify spore presence and identify any residual vegetative bacteria in the sample. At least 100 cells from each sample were observed and counts taken for the number of spores, spores-in-mother cells, and vegetative cells.

### Phage Generation

Phages specific for *P. larvae* were previously isolated and confirmed to infect and lyse only *P. larvae* (Brady et al., 2017; Stamereilers et al., 2018) and not *B. laterosporus* (Brady et al., 2017; Berg et al., 2018; Brady et al., 2018). Phages were tested on field isolates of *P. larvae.* Each field isolate for phage characterization was identified as *P. larvae* by confirming gram test, catalase test, 16s sequencing, and by KAT PCR and ERIC PCR. Primers and citations for these methods are indicated in Table 1. Protocols are published in our previous works (Brady et al., 2017; Berg et al., 2018; Brady et al., 2018). The phage, PL.Ph-1, was previously isolated from a dead feral beehive in Utah that appeared to have died from AFB. PL.Ph-1 forms lytic plaques on the laboratory standard *P. larvae*, ATCC 9545, as well as on 55 of 59 verified field isolates of *P. larvae* as previously reported (Brady et al., 2017). Phage lysate of PL.Ph-1 [the phage also indicated as phage 1 in Brady et al. (2017)] was prepared for these studies by reconstituting a freezer stock sample of the phage in 500 μL of overnight *P. larvae* and plating the solution in 1/2 × PBHI top agar and left to incubate at 37°C. After 24 h, visible plaques were selected from the plate, suspended in 25 ml of 1/2 × PBHI broth to which was added 1 × 10^6^ CFU of *P. larvae* and incubated, shaking at 37°C. After 16 h the lysate was filtered through a 0.22 μM filter (VWR, Radnor, PA, United States). Lysate concentration was determined using standard titration techniques as previously described to determine plaque forming units (pfu) per ml (Brady et al., 2017). Titered lysate of PL.Ph-1 was used for all studies in this paper.

**TABLE 1:** Primers for PCR identification of *P. larvae* field isolates used to challenge *P. larvae* phages.

Primer	Sequence	Results	References
16s 27-forward	5′-AGAGTTTGATCMTGGCTCAG-3′	16s rRNA universal primer	Lane, 1991
16s 907-reverse	5′-CCGTCAATTCMTTTRAGTTT-3′		
KAT-Forward	5′-ACAAACACTGGACCCGATCTAC-3′	Generates a band only if *P. larvae* ERIC-1 or ERIC-2 genotype	Alippi et al., 2004
KAT-Reverse	5′-CCGCCTTCTTCATATCTCCC-3′		
ERIC1-Forward	5′-ATGTAAGCTCCTGGGGATTCAC-3′	Generates a series of bands used to identify *P. larvae* types	Versalovic et al., 1994
ERIC1-Reverse	5′-AAGTAAGTGACTGGGGTGAGCG-3′		

### Phage Binding Detection by FITC Stain and Flow Cytometry

Unconjugated FITC was added to a high titer phage lysate (10^11^ pfu) suspended in 1× HEPES solution (pH 7.4) to obtain a concentration of 31.25 μg/ml FITC and was allowed to incubate for 1 h. The high titer lysate was ultracentrifuged at 25,000 × *g* for 1 h to pellet the phages. The supernatant containing unbound FITC was poured off and the pellet resuspended in HEPES solution to a FITC concentration of 15.625 μg/ml to avoid background staining of bacteria as indicated in the results.

For flow cytometry analysis, bacterial samples were loaded into a 96-well plate containing approximately 5 × 10^4^ CFU in each well. Each well received 200 μL of FITC-labeled phages. Cell fluorescence was measured by a Cytoflex S flow cytometer and a minimum of 50,000 cells were counted per sample. Three experiments were run on separate weeks with three replicates for each sample in each experiment.

### Flow Cytometry Data Analysis

Beckman Coulter CytExpert software was used to analyze the flow cytometry data collected on the Beckman Coulter Cytoflex S flow cytometer. Three gates were individually set using unstained samples of each bacterial type using channels for Forward Scatter (FSC), Side Scatter (SSC) and FITC. The gates were set on FSC × SSC to exclude debris, FSC-HxFSC-A to isolate single cells, and on the FITC channel to identify positive or negative samples.

### Phage Binding Detection by Electron Microscopy

Vegetative *P. larvae* and *P. larvae* spores (5 × 10^5^ CFU) were resuspended in 1 ml of 3 × 10^9^ pfu/ml high titer lysate and allowed to incubate for 1 h. The phage-treated spores were pelleted at 8,000 rpm for 6 min. The supernatant was poured off and the pellet was resuspended in 40 μL of 1× HEPES solution.

Phage/spore samples were incubated with formvar coated copper grids for 60 s and then incubated with 50 μL of 2% uranyl acetate (pH 7) for 60 s. Moisture was wicked away from the grids and then allowed to air dry prior to imaging. Electron micrographs were taken by the BYU Microscopy Center on a Verios STEM machine (Abràmoff et al., 2004).

### Phage Binding and Viability Detection Using the Brady Binding Assay

The Brady Binding Assay was developed in our lab to identify the ability of a phage to reversibly bind to a test material (such as a spore or unrelated bacteria) and to remain viable against its original target. In brief, the assay is setup by incubating the phage with the test bacterium, transferring the sample onto a filter, and then rinsing the trapped bacteria to remove un-bound phages. The trapped, rinsed, bacteria are incubated with bacteria of the original phage target and a standard plaque assay is done. Brady binding assays for this study were setup as follows: overnight cultures of *P. larvae* ATCC 9545, *B. laterosporus* field isolate B-2, *Sinorhizobium meliloti* strain B100, and *P. larvae* ATCC 9545 spores were each diluted to 10^4^ CFU/ml. The Brady binding assay was setup each time using an MOI of 10^4^ with each sample in triplicate. The bacteria were pelleted, supernatant discarded, and the pellets resuspended in 1 ml of phage lysate at a titer of 10^8^ pfu/ml, control samples of phage lysate were resuspended in 1 ml of sterile 1/2 × PBHI broth without bacteria, and all samples were incubated for 30 min at room temperature. Each solution was filtered using single-use 0.22 μM vacuum filter to remove all bacteria. The filters were then rinsed with 1 L of 1× phosphate buffered solution (PBS) to release any phages that were not bound to bacteria. The filters were removed, placed in tubes containing 1 ml of 1/2 × PBHI broth, and vortexed for 1 h to dislodge bacteria from the filter. After vortexing, a standard plaque assay was setup using 100 μL of sample (or diluted sample) to incubate 5 or less minutes with 500 μL of overnight *P. larvae* ATCC 9545, plate in 1/2 × PBHI top agar, and incubate overnight. The resulting plaques were counted and data reported as the average ± standard error of the mean. Data was converted into percentages to compare with flow cytometry results by first dividing the average number of plaques from the positive control group in the Brady assay by the percentage of FITC-positive cells in the corresponding positive control group from flow cytometry. The resultant number was used as the denominator to convert the average number of plaques from each group in the Brady assay into a percentage.

### Statistics

Data were analyzed using SAS software (SAS Institute Inc., Cary, NC, United States) and the Mixed Procedure method to generate *p*-values, standard deviation, standard error and to determine statistical significance (for Figures 4, 8). For direct count statistics in 3.1, we used Jeffery’s 95% confidence interval (Brown et al., 2001) for binomial proportions. For all experiments α = 0.05.

## Results

### Phage PL.Ph-1 Was Specific for *P. larvae* and the *P. larvae* Spores Prepared for Binding Studies Were Pure and Viable

**FIGURE 1:**
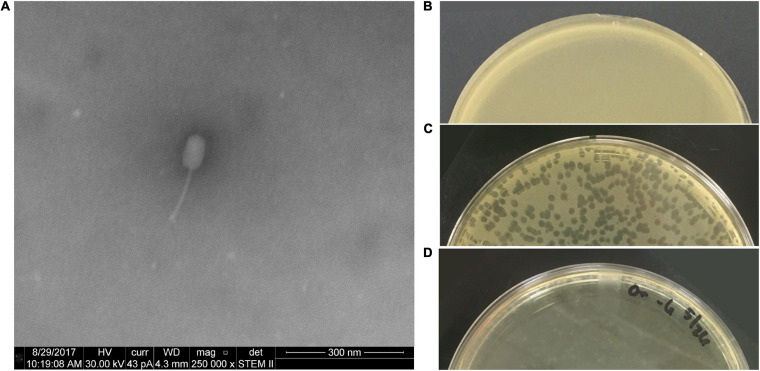
PL.Ph-1 is a siphovirus with a prolate head **(A)** capable of forming plaques on *P. larvae*. A control plate of ATCC 9545 **(B)**, plaques of PL.Ph-1 **(C)**, and a fully lysed plate **(D)** demonstrate the lytic ability of the phage.

Figure 1 includes an electron micrograph of phage PL.Ph-1 and images of plates demonstrate the lytic nature of the phage used in these studies. PL.Ph-1 is a siphovirus with a prolate head and is the same phage labeled #1 in studies by Brady et al. (2017) which was used in the phage cocktail for hive treatments in that same paper. This phage was selected for further study because, as previously reported, it was the only phage that readily formed plaques on more than 90% of the field isolates to which it was challenged compared to the other 38 phages that formed plaques on an average of only 39.4 ± 2.4% of field isolates (Brady et al., 2017). Challenge of the PL.Ph-1 phage on multiple strains of *B. laterosporus* did not yield plaques, which was not surprising since none of the other 38 isolated *P. larvae* phages formed plaques on *B. laterosporus* when tested in our lab.

In order to study phage binding to spores, the *P. larvae* spores to be used for the study were rigorously tested to ensure purity of the prepared spores. A pure spore sample is free of vegetative cells and composed only of spores with few spores-in-mother cells. No vegetative cells were identified in the spore samples using this method and free endospore comprised 92 ± 2.1% of the cells. The samples contained 8.0 ± 2.6% of spores that had not released from their mother cell.

**FIGURE 2:**
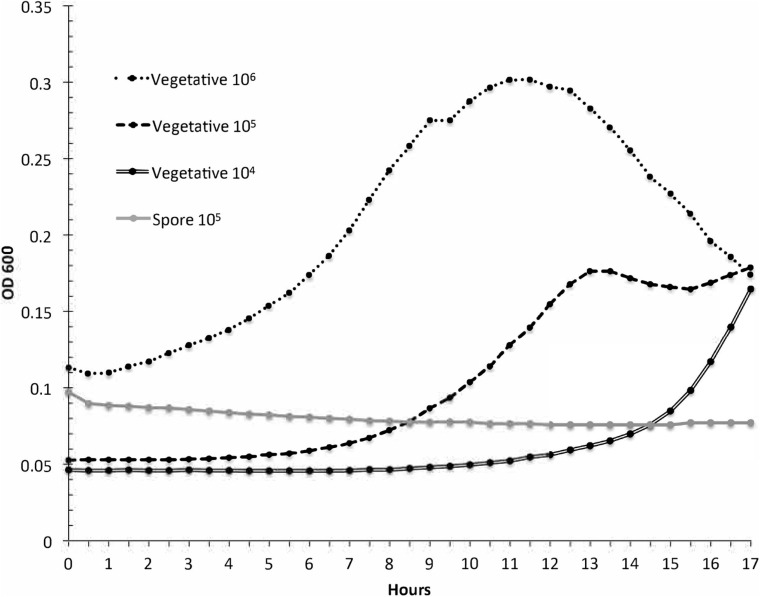
*Paenibacillus larvae* growth curves are used to test spores for contaminant vegetative cells. The optical density was measured for three dilutions of vegetative *P. larvae* and for *P. larvae* spores during a 17-h incubation in broth. The resulting curves indicate that the spore sample did not contain detectable vegetative bacteria.

Spore samples were incubated in 1/2 × PBHI broth to further confirm the spore purity of the samples. The purpose of this study was to verify that the spore sample used for subsequent studies did not contain vegetative cells and would not germinate spores in the time span of the experiments. Results of this study are presented in Figure 2. Positive controls of vegetative bacteria were incubated with starting concentrations of 10^6^, 10^5^, and 10^4^ CFU/ml. Spores had an approximate concentration of 10^5^CFU/ml. The optical density of the spore samples did not change significantly over 17 h in comparison to the vegetative *P. larvae* samples at 10^6^, 10^5^, and 10^4^ CFU/ml over the same amount of time. Spores generated from strain ATCC 9545 do not germinate in 1/2 PBHI liquid media without at least previously being heat activated for 30 min at 70°C (Alvarado et al., 2013); therefore, any increase in optical density of an incubated sample in our studies would result from vegetative bacterial growth in the sample and not from spore germination. These data indicate that the spore samples did not contain any detectable amounts of vegetative bacteria. All studies were conducted with 17 or fewer hours of incubation.

The ethanol-wash treatment used to prepare spores was also tested on vegetative *P. larvae* to verify that the ethanol treatment had killed any surviving vegetative cells in the spore samples. No colonies formed from ethanol wash samples. Spore samples were plated for germination to ensure their viability. After a 48-h incubation, colonies formed on spore-inoculated plates and the colonies were confirmed to be *P. larvae*.

### Flow Cytometry Studies Indicated That Phages Bind to Spores and Related Bacteria

**FIGURE 3:**
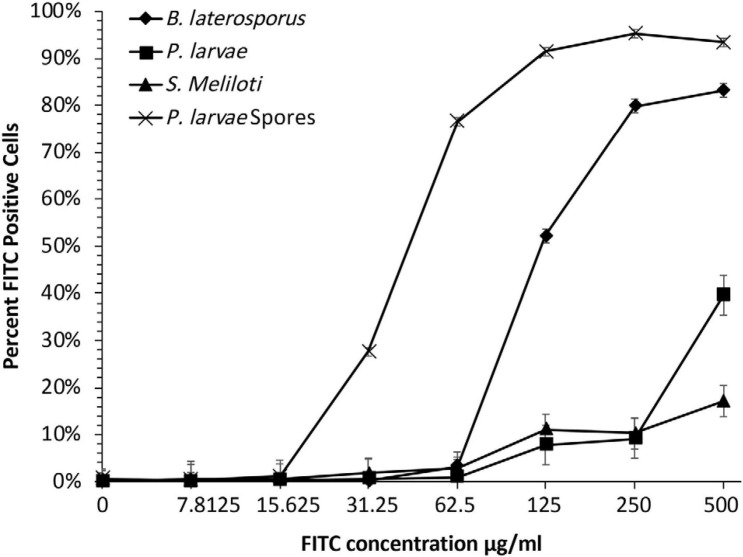
For detection of FITC-stained phages, FITC must be optimized to a concentration below where bacteria will absorb detectable stain. Bacterial samples were dosed with seven FITC concentrations to determine background-fluorescence of bacteria. Samples were compared to untreated groups to establish what concentration of FITC caused background staining. Bacterial fluorescence was detected at doses of 15.625 μg/ml or higher.

Use of flow cytometry to measure phage binding has not previously been reported. We anticipated that phage binding could be quantified using flow cytometry because others have demonstrated that phages could be stained with FITC fluorochrome and then visualized by confocal microscopy (Kelly et al., 2006; Puapermpoonsiri et al., 2010). A flow cytometer takes a reading of each cell in a suspension and reports the fluorescence intensity of each individual cell. Flow cytometry can produce rapid, quantitative results from thousands of individual bacterial cells in each sample while ignoring free-floating phages. We first needed to establish the highest concentration of FITC stain that would not generate background staining on the bacteria. Bacterial samples were incubated with different concentrations of FITC stain to be used for phage staining (Figure 3). These results indicated that the bacteria should not be exposed to FITC solutions of 31.3 μg/ml or greater, otherwise background staining of the bacteria would be observed in the flow data and could complicate interpretation in samples containing stained phages. The experiment proceeded with phages in a solution containing 15.6 μg/ml of FITC to be incubated with the test bacteria.

**FIGURE 4:**
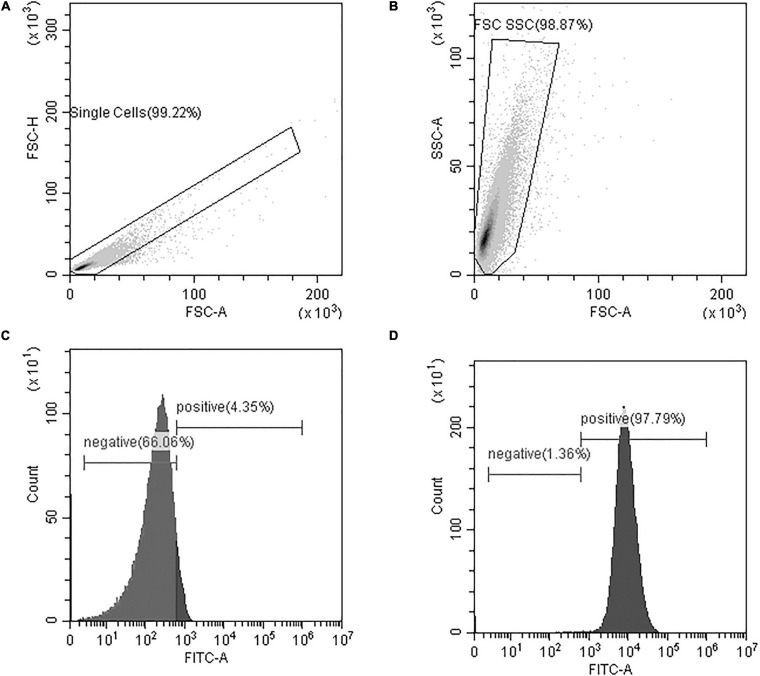
Flow cytometry data analysis included **(A)** a gate to isolate single cells from the sample based on a plot of Forward Scatter Area and Height readings (FSC-A and FSC-H, respectively) **(B)** a gate to eliminate small and large debris based on Forward and Side Scatter Area readings (FSC-A and SSC-A, respectively), and **(C,D)** a histogram of the gated cells with FITC fluorescence-intensity on the x-axis and the number of cells on the y-axis. The FITC histograms yield peaks in the positive or negative regions of the plot and percentages are reported. Representative samples are shown for **(C)** a FITC-negative sample, and **(D)** a FITC-positive sample. At least 50,000 cells were analyzed from each sample.

As depicted in Figure 4, data analysis of flow cytometry results utilized a series of three plots to identify whether or not the fluorescent phages bound to bacteria. The first two plots were used to select the population to analyze (Figures 4A,B) and the third plot reported the number of cells with each intensity of FITC staining (Figure 4C is a negative sample and Figure 4D is a positive sample). On the histogram of the FITC reading, cells that are positive indicate bacteria with phages attached.

**FIGURE 5:**
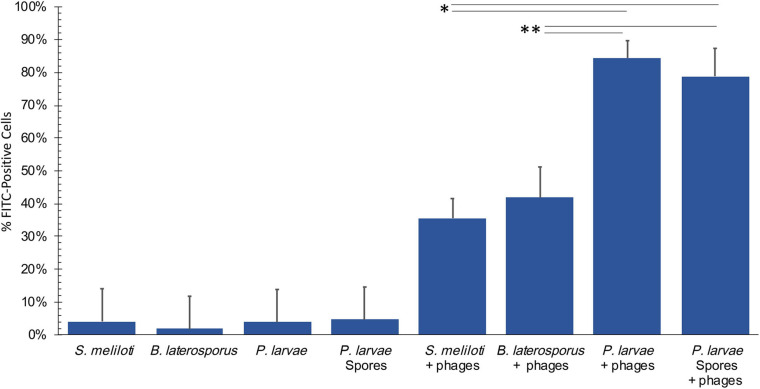
Flow cytometry results detect phages bound to bacteria and spores. Negative controls (first four columns), were low, and all samples with phages were statistically significant compared to the negative controls excluding *S. meliloti*. Statistically significant data is indicated with asterisks. See Table 2 for averages and *p*-values from the statistical analysis of samples with phages.

FITC-stained phages were mixed with vegetative *P. larvae*, *B. laterosporus*, *S. meliloti*, and spores of *P. larvae*. The average percentage of FITC-positive bacterial cells for each is reported in Figure 5. *P. larvae*, *B. laterosporus*, and *P. larvae* spores treated with labeled phages are significantly different (*p* < 0.0001) from untreated samples where *S. meliloti* treated with phages did not have a statistical difference from an untreated sample (*p* = 0.3725). Table 2 contains the *p*-values for statistical analysis of data from the last four columns in Figure 5 to compare bacterial sample containing phages with each other. Phage binding to *S. meliloti* and to *B. laterosporus* is significantly different compared to phage binding to *P. larvae*, regardless of whether it is vegetative or spore forms of *P. larvae.* Phage binding to *S. meliloti* is not statistically different from phage binding to *B. laterosporus*. Phage binding is also not statistically different when comparing vegetative and spore forms of *P. larvae* to each other. These data support the hypothesis that *P. larvae* phages bind to spores.

**TABLE 2:** Average percentage of FITC-positive cells by flow ctyometry.

	Average % of FITC-positive cells	*S. meliloti*	*B. laterosporus*	*P. larvae*	*P. larvae* spores
*S. meliloti*	29.1 ± 5.7%	–	0.8127	0.0001	0.0001
*B. laterosporus*	41.8 ± 6.5%		–	0.0004	0.0056
*P. larvae*	84.4 ± 5.7%			–	0.9979
*P. larvae* spores	78.8 ± 6.5%				–

**P*-values are from statistical results comparing bacterial sample challenged with phages.*

### Electron Microscopy Visually Confirmed That Phages Attach to Spores

**FIGURE 6:**
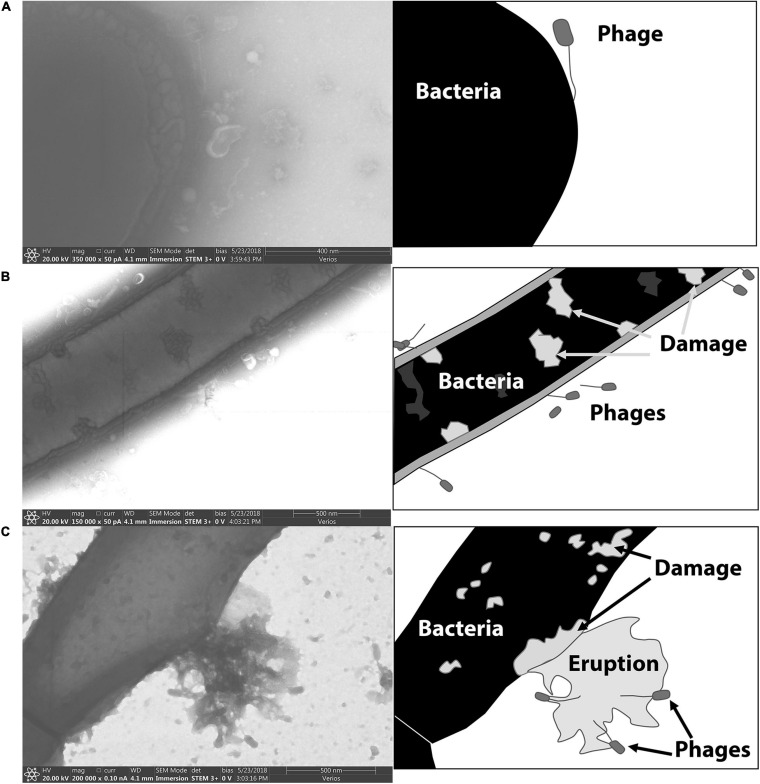
STEM images of phages bound to the vegetative form of *P. larvae*. Cartoon depictions in the panels to the right of each electron micrograph include labels of notable items. **(A)** A phage is attached to the end of a vegetative *P. larvae* bacterium. **(B)** Several phages are visibly attached to the sides of vegetative *P. larvae.* **(C)** A vegetative *P. larvae* bacterium is undergoing lysis after productive phage infection.

Electron microscopy was used to visualize phage-binding to spores. Figure 6 includes electron micrographs of vegetative *P. larvae* incubated with phages. Phages were observed in horizontal and vertical positions with respect to the bacterium. Most commonly, the phages were attached by the end of the tail fiber to the side of the vegetative bacterium. Visible damage occurred to the bacterial wall upon infection, and late-stage infection was observed as an eruption from the bacterial wall.

**FIGURE 7:**
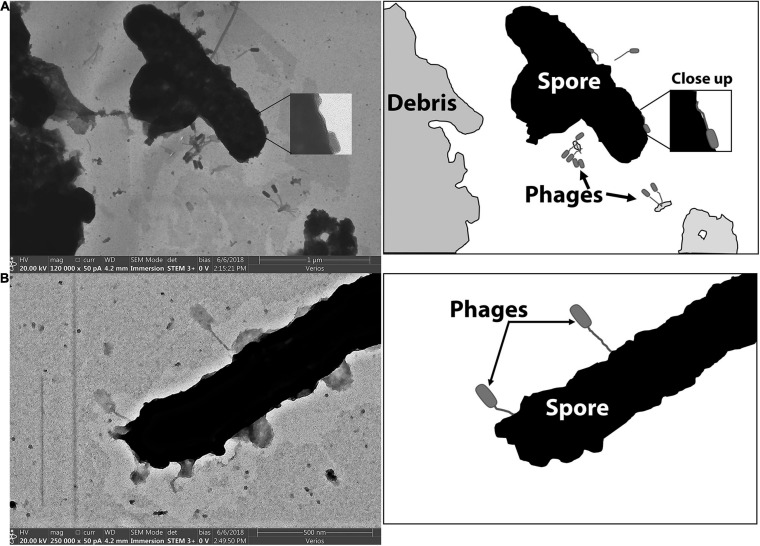
STEM images of phages bound to the spore forms of *P. larvae*. Cartoon depictions in the panels to the right of each electron micrograph include labels of notable items. **(A)** Phages are attached to a mother-in-spore of *P. larvae.* **(B)** Phages are attached to a fully-formed spore of *P. larvae.*

Figure 7 includes electron micrographs of *P. larvae* spores incubated with phages. Phages were observed in horizontal and vertical positions on the spores. Spores that had not yet released from the mother cell were equally prone to have phages attached, and the phages appeared equally able to attach to the spore side as to the mother cell side (Figure 6A). Phages were observed in both horizontal and vertical positions on spores. Some phages clearly attached to the spore by the end of the tail fiber (Figure 6B). Phages appeared to be attached in higher abundance on vegetative cells than on spores as observed in electron micrographs. Our imaging methods did not distinguish between empty or full capsids, so no interpretation can be made regarding whether or not a phage injected its DNA into a spore or not.

### Results of the Binding Assay Demonstrated That Phages Reversibly Bind to Spores and That the Phages Are Still Infectious

**FIGURE 8:**
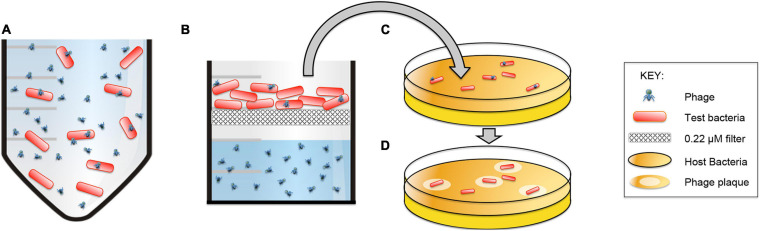
The Brady Binding Assay detects viable phages that directly infect and/or reversibly-bind to bacteria. **(A)** Phages are incubated with test bacteria. **(B)** Bacteria are caught on a filter and heavily rinsed with PBS to remove non-bound phages. **(C)** Bacteria are removed from the filter and plated with their host bacteria to incubate for 24 h. **(D)** Viable phages form visible plaques on the host bacteria which indicates that direct infection or reversible binding has occurred between the phages and the test bacteria.

The Brady Binding Assay includes a short incubation period with phages and a long rinse of the bacteria prior to a plaque assay (Figure 8). Since the thoroughly rinsed bacteria are used as the source of phages for the plaque assay, plaques indicate that binding to the challenge bacteria occurred during the initial short incubation period. Phages can produce plaques in two ways: (1) the phages are able to productively infect challenge bacteria during the first step and then produce plaques on their intended host in the plaque assay, or (2) the phages can exhibit reversible binding wherein phages bind to the challenge bacteria in the first step and release to infect and produce plaques on their intended host in the plaque assay.

**FIGURE 9:**
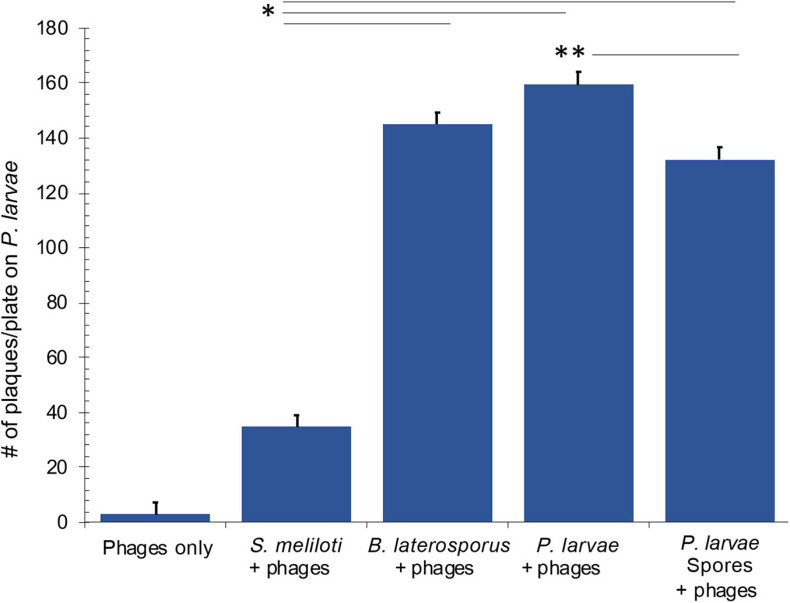
Results of the Brady assay indicate that phages bind to vegetative and spore samples of *P. larvae*, and to vegetative *B. laterosporus*. All samples where phages were challenged with bacteria were statistically different from the phages only control. Statistically significant data between test bacteria samples are indicated with asterisks. See Table 3 for averages and *p*-values from the statistical analysis.

Results of the Brady assay are presented in Figure 9 and Table 3. The “phages only” control indicates the resultant number of phages that become mechanically trapped on the filter during the Brady assay. A statistically significant number of plaques formed from all test bacteria samples compared to the phage only samples. By comparison, *P. larvae* spores formed plaques significantly higher than the unrelated *S. meliloti* and statistically lower than vegetative *P. larvae.* The number of plaques from *B. laterosporus* was surprisingly not statistically different from that of vegetative *P. larvae*, nor was it statistically different from that of *P. larvae* spores (α = 0.01). These results indicate that *P. larvae* phages bind with significant abundance to vegetative *P. larvae, P. larvae* spores and to vegetative *B. laterosporus*.

**TABLE 3:** Average plaque counts from the Brady Binding Assay.

	Average plaque counts	*S. meliloti*	*B. laterosporus*	*P. larvae*	*P. larvae* spores
Phages only	3 ± 10	<0.0001	<0.0001	<0.0001	<0.0001
*S. meliloti*	35 ± 9	–	<0.0001	<0.0001	<0.0001
*B. laterosporus*	145 ± 9		–	0.1925	0.2494
*P. larvae*	159 ± 10			–	0.0018
*P. larvae* spores	132 ± 9				–

**P*-values are from statistical analysis comparing phage binding in each bacterial sample challenged with phages.*

### Comparative Results of Flow Cytometry Detection and the Brady Binding Assay Allow Interpretation of the Phage-Binding Ability of PL.Ph-1

Results from the Brady assay were converted into percentages so that the data could be compared between the two quantitative methods used in our research. Table 4 provides a comparison between flow cytometry data and Brady assay results and summarizes a proposed interpretation of these data.

**TABLE 4:** Comparison of phage binding capability data from results of Flow cytometry and the Brady Binding Assay.

	% binding by Flow cytometry	% binding by Brady assay	Phage-binding ability	Fluorescence intensity for Flow cytometry detection	Percentage of cells with phages attached	Reversibility of binding
*S. meliloti*	29.1 ± 5.7%	18.4 ± 2.2%	Very low number of phages per bacterium	Dim to undetected	Low percentage of cells have phages	Low reversibility?
*B. laterosporus*	41.8 ± 6.5%	76.8 ± 2.2%	Low number of phages per bacterium	Dim to undetected	High percentage of cells have phages	High reversibility
*P. larvae*	84.4 ± 5.7%	84.4 ± 2.5%	Many phages per bacterium	Bright	High percentage of cells have phages	Moderate reversibility?
*P. larvae* spores	78.8 ± 6.5%	70.0 ± 2.4%	Moderate number of phages per bacterium	Bright	High percentage of cells have phages	Moderate reversibility

## Discussion

The body of evidence from our data supports that *P. larvae* phages do, indeed, bind to *P. larvae* spores. Differences in results from flow cytometry and from the Brady binding assay data should come under careful scrutiny considering that, while still supportive of our hypothesis, these two detection methods yielded different results. As presented in Table 4, we believe that these data evidence important information about what each methods is able to detect, as well as the reversibility, and potentially the evolutionary survival strategies of the phages.

A few factors should be considered when interpreting flow cytometry data. The phages were stained with FITC for detection of phage binding to bacteria. The more phages that attach to an individual bacterium, the brighter the fluorescence of that bacterium. However, we did not report the brightness of the cell, but rather the total number of cells that are above a threshold of brightness. This means that the cutoff point, as indicated in Figures 4C,D, will count cells as positive, as long as enough phages are attached to fluoresce the bacteria above the cutoff. Based on the fact that more plaques were formed in the Brady assay for *B. laterosporus* than were detected by flow cytometry, our cutoff point for flow cytometry excludes bacteria that may have a very low number of phages per bacterium.

Initially we posited that, since spores are inactive, phages would not permanently bind to spores, but could reversibly bind in order to release from the spore and infect vegetative bacteria. Since the Brady assay does not support activation of spores, plaques produced from the spore sample must indicate reversible binding and must further indicate that the phages are still active for infection after binding and releasing from the spores. The results are supportive of this interpretation because spore and vegetative samples were not different by flow cytometry, but were, statistically, slightly lower in the spore sample by Brady assay. The statistically lower number of plaques in the spore sample by Brady assay likely represent spores that retain the bound phages. Some phages are expected to remain on spores for better survival of the phages. Since spore germination in a bee infection only occurs inside the larval gut, phages that are attached to spores must have survival and transmission strategies to eventually gain access to vegetative bacteria of their host.

The plaque assay used as part of the Brady assay technique generates a single plaque regardless of whether the bacterium in that location carried a high or low number of phages. This assay, therefore, provides a good view of what percentage of cells have phages attached, but does not represent how many phages were attached to a single bacterium. Furthermore, if the phage can infect the test bacterium, as is the case with vegetative *P. larvae*, then the Brady assay results cannot reflect whether phage binding is reversible since the phages can productively infect and form a plaque without having to reversibly bind. Data in Table 3 confirms that the results of the Brady assay directly reflect that of Flow cytometry for vegetative *P. larvae.*

For our studies, we intentionally did not heat activate spores so that the spores would not be able to germinate during an assay. Non-germinating spores would ensure that any plaques that formed on the vegetative lawn in a Brady assay would be from a surface release of the phages from the spores rather than an infection of the spores themselves. In like manner, our study used phages that cannot productively infect *B. laterosporus* or *S. meliloti*, so that any plaques formed on the *P. larvae* vegetative lawn would again be a result of binding and subsequent surface release of the phages from these bacteria rather than an infection of them. Non-binding phages were rinsed away prior to the challenge bacteria being placed with the vegetative *P. larvae*. We conclude that the observed plaques must come from reversible binding.

Results from *B. laterosporus* indicate that *P. larvae* phages bind to this non-host bacterium, but can readily reverse binding and productively infect their *P. larvae* host. The high number of plaques observed from this sample in contrast to the low flow cytometry counts, indicates that many *B. laterosporus* bacteria have a small number of phages attached that can reversibly bind and subsequently infect *P. larvae*. For instance, a single phage on *B. laterosporus* would be very unlikely to be detected by flow cytometry, but would yield a plaque in the Brady assay. Reversible binding may be a mechanism of survival of the phages but does not preclude other mechanism, such as capture and protection of phage DNA within bacterial spores of the host bacterium (Silver-Mysliwiec and Bramucci, 1990; Walter, 2003; Sonenshein, 2016; Gabiatti et al., 2018). The increased binding and reversible capability of *P. larvae* phages on a different bacterial species than the phages’ host and which bacterial species resides in the same niche space as the phages’ host (in this case *B. laterosporus*) is a newly identified mechanism for phage survival.

*Brevibacillus laterosporus* is a firmicutes bacterium distinctly separate from *P. larvae* but recently identified as having significant genetic similarities (Berg et al., 2018) in addition to their both residing in beehives. We did not anticipate the high numbers of plaques generated from *B. laterosporus* bacteria in the Brady assay because *P. larvae* phages used for this study do not productively infect *B. laterosporus. B. laterosporus* has been debated as to whether it is a commensal or a pathogenic bacterium in honeybees (Charles and Nielsen-LeRoux, 2000; Ruiu et al., 2012, 2014; Ruiu, 2013; Bashir et al., 2016; Marche et al., 2016; Mura and Ruiu, 2017). Studies indicate that *B. laterosporus* at least increases in prevalence during an *P. larvae* infection in the hive (Alippi et al., 2002). The presence of *B. laterosporus* during AFB infections led to the development of a bystander phage therapy for AFB (Brady et al., 2018), albeit the phage for bystander treatment was a *B. laterosporus* phage. Since the *P. larvae* lifecycle includes spore transmission, we anticipated that phages must have a mechanism of survival between the spore stage and the next germination. While we hypothesized that this survival would be due to spore binding, we did not anticipate an alternative hypothesis that *P. larvae* phages bind to *B. laterosporus* in a survival strategy, as our data indicate. While *B. laterosporus* is able to sporulate, we have not yet explored whether or not *P. larvae* phages can also bind to *B. laterosporus* spores. Studies with *B. laterosporus* spores as well as other bacterial species will be interesting to explore for the level of phage binding. Since *B. laterosporus* is also found in beehives and is even more often found alongside *P. larvae*, we posit that this phage binding is an evolutionary survival strategy of the phages.

Electron microscopy images visually demonstrated that these phages bind to the surface of spores in various orientations. Other researchers used cryotomography of T4 phages to reveal different phage orientations during the infection process (Leiman et al., 2010; Hu et al., 2015; Taylor et al., 2016). Their results suggest that long tail fibers first bind to target bacteria and generate pressure that triggers the release of short tail fibers from the baseplate. The short tail fibers from the baseplate bind to specific receptors on the surface of the bacterium, which in turn erects the phage and triggers the injection of DNA into the cell (Leiman et al., 2010; Vinga et al., 2012; Hu et al., 2015; Taylor et al., 2016). Both horizontal and vertical binding was apparent in our electron microscopy samples of both vegetative and spore *P. larvae*. If the erect phages have bound and injected DNA into the cell, then *P. larvae* phages may be able to directly infect *P. larvae* spores similar to other spore-infecting phages (Fu et al., 2011). The alternative phage orientation on the bacterium and spores in our images may indicate differences between reversible and irreversible binding, and/or may indicate that DNA injection occurs with both the vegetative and spore forms of *P. larvae*. Our staining and imaging methods do not allow distinction of whether or not DNA was injected.

In our samples, approximately 8% of spores had not exited the mother cell. We anticipated that vegetative cell receptors would remain on the mother cell side to which phages could bind. By electron microscopy, phages were observed attached to both the spore side and the mother side of these spores. The quantitative data well exceeded that of 8% of phage-positive spores using flow cytometry and by the Brady assay. Further, electron microscopy clearly captured phages attached to mature spores, indicating that phage binding can occur on a fully formed *P. larvae* spore. Since the phage-bound spores were bright by flow cytometry, we anticipate that a moderate to high number of phages attached to each spore, and the majority of spores detached at least one phage in order to form plaques to result in such a high number of plaques from these samples. Whether or not the phages that remained on the spores had already or could infect a spore to yield a productive infection upon germination is yet to be determined.

Our results directly support the hypothesis that spore-binding phages may prevent reinfection from spores of a recovered beehive. In field studies of AFB-infected hives treated with *P. larvae* phages, not only did beehives recover from AFB in less than 2 weeks but the hives also did not become re-infected for the following 6 months of observation (Brady et al., 2017). This contrasts with the fact that bystander phage therapy, which uses phage-induced toxins capable of killing vegetative *P. larvae*, can cure active AFB within 2 weeks but AFB re-emerges within 1 month of treatment (Brady et al., 2018), and furthermore that antibiotic treatment of hives also experience re-emergence of AFB within months of clearing an active infection using antibiotics (Alippi, 1996; Alippi et al., 1999; Brady et al., 2017). These studies indicate that if a hive is treated to kill only vegetative bacteria and spores are not neutralized, then a re-infection will occur. Evidence of spore binding as presented in this report provides a mechanistic explanation for the success of the phages used in the hive treatment studies. Reversible binding of *P. larvae* phages on spores and on *B. laterosporus* increases the likelihood of the phages encountering vegetative *P. larvae* when spores germinate or when *B. laterosporus* expands as a secondary infection to AFB. *B. laterosporus* may be a commensal or a pathogenic bacteria in honeybees (Alippi et al., 2002). As a commensal, reversible binding to this bacteria is a viable mechanism for retaining phages within the hive for protection against reinfection by *P. larvae*. Alternatively, phages that can attach to *P. larvae* spores may directly infect or may release from and infect newly germinated vegetative *P. larvae*.

Although this work did not show that the phages directly kill spores, other phages have been identified that do (Walter, 2003; Fu et al., 2011). By hunting for phages that specifically bind to and/or destroy spores, phage cocktail therapies against sporulating bacterial species will likely have a greater potential for functionality with a possibility of preventing recurrent infections caused by spores. The results presented in this report are the first to demonstrate that *P. larvae* phages can be bind to both *P. larvae* spores and to vegetative *B. laterosporus*.

## Data Availability Statement

The raw data supporting the conclusions of this article will be made available by the authors, without undue reservation.

## Author Contributions

TB, CF, and SH: conceptualization. TB, JW, CF, and CR: lab work. TB, JW, CR, DE, and SH: analysis. TB: writing – original draft preparation. CR, DB, and SH: writing – review and editing. DB and SH: supervision. SH: project administration. TB and SH: funding acquisition. All authors contributed to the article and approved the submitted version.

## Conflict of Interest

The authors declare that the research was conducted in the absence of any commercial or financial relationships that could be construed as a potential conflict of interest.
